# The e-mental health treatment in Stockholm myocardial infarction with non-obstructive coronaries or Takotsubo syndrome study (E-SMINC): a study protocol for a randomised controlled trial

**DOI:** 10.1186/s13063-022-06530-3

**Published:** 2022-07-26

**Authors:** Erik M. G. Olsson, Fredrika Norlund, Elisabet Rondung, Sophia M. Humphries, Claes Held, Patrik Lyngå, Jonas Spaak, Örjan Sundin, Runa Sundelin, Philip Leissner, Lena Kövamees, Per Tornvall

**Affiliations:** 1 Department of Women’s and Children’s Health, Uppsala University, Uppsala, Sweden; 2 Department of Psychology and Social Work, Mid Sweden University, Östersund, Sweden; 3 Department of Medical Sciences, Cardiology, Uppsala Clinical Research Center, Uppsala University, Uppsala, Sweden; 4 Department of Clinical Science and Education, Karolinska Institutet and Department of Cardiology, Södersjukhuset, Stockholm, Sweden; 5 Department of Clinical Sciences, Danderyd University Hospital, Karolinska Institutet, Stockholm, Sweden; 6 Swedish Heart and Lung Association, Stockholm, Sweden

**Keywords:** MINOCA, Takotsubo syndrome, Stress, Anxiety, Cognitive behavioural therapy, Internet-based intervention, Randomised controlled trial

## Abstract

**Background:**

In the aftermath of a myocardial infarction with non-obstructive coronary arteries (MINOCA) or Takotsubo syndrome (TS), patients commonly express high levels of stress and anxiety. Current treatment alternatives rarely address these issues.

**Methods:**

The study is a randomised controlled trial, where 90 patients with a discharge diagnosis of MINOCA or TS who also report symptoms of stress or anxiety will be randomised 2–6 weeks after their cardiac event. The treatment consists of 10 weeks of Internet-based cognitive behaviour therapy (CBT) and starts immediately after randomisation for the treatment group. The control group receives usual care. Main outcomes are symptoms of anxiety measured with the Hospital Anxiety and Depression scale, anxiety subscale, and perceived stress measured with the Perceived Stress Scale, 14-item version, 10 weeks after randomisation. Secondary measures include cardiac specific anxiety, symptoms of post-traumatic stress, quality of life, cortisol measured in hair and physiological stress responses (heart rate variability, blood pressure and saliva cortisol) during a stress procedure. Ten weeks after randomisation, the control group will also receive treatment. Long-term follow-up in the self-report measures mentioned above will be conducted 20 and 50 weeks after randomisation where the total group’s development over time is followed, and the groups receiving intervention early versus late compared.

**Discussion:**

At present, there are no randomised studies evaluating psychological treatment for patients with MINOCA or TS. There is an urgent need for treatment alternatives aiming at relieving stress and anxiety considering the high mental stress and anxiety levels observed in MINOCA and TS, leading to decreased quality of life. CBT aiming at reducing mental stress has been shown to be effective regarding prognosis in patients with coronary artery disease. The current protocol describes a randomised open-label controlled trial evaluating an Internet-based CBT program for reduction of stress and anxiety in patients with increased mental stress and/or anxiety with a discharge diagnosis of either MINOCA or TS.

**Trial registration:**

ClinicalTrials.govNCT04178434. Registered on 26 November 2019

## Administrative information

Note: the numbers in curly brackets in this protocol refer to SPIRIT checklist item numbers. The order of the items has been modified to group similar items (see http://www.equator-network.org/reporting-guidelines/spirit-2013-statement-defining-standard-protocol-items-for-clinical-trials/).

**Table:** 

Title {1}	The e-mental health treatment in Stockholm myocardial infarction with non-obstructive coronaries or Takotsubo syndrome study (E-SMINC), a study protocol for a randomized controlled trial
Trial registration {2a and 2b}.	Clinicaltrials.gov NCT04178434
Protocol version {3}	Version 2:1 September 2021
Funding {4}	Vetenskapsrådet (Swedish Medical Research Council) Grant 2018-02655, Hjärt-Lungfonden (Swedish Heart and Lung Foundation) Grant 20200221, Riksförbundet HjärtLung (The Swedish Heart and Lung Association) Grant FA 2019:41 and 2020:21.
Author details {5a}	Erik MG Olsson, Department of Women’s and Children’s Health, Uppsala University; Fredrika Norlund, Department of Women’s and Children’s Health, Uppsala University; Elisabet Rondung, Department of Psychology and Social Work, Mid Sweden University; Sophia M Humphries, Department of Women’s and Children’s Health, Uppsala University; Claes Held, Department of Medical Sciences, Cardiology, Uppsala Clinical Research Center, Uppsala University; Patrik Lyngå, Department of Clinical Science and Education, Karolinska Institutet and Department of Cardiology Södersjukhuset; Jonas Spaak, Department of Clinical Sciences, Danderyd University Hospital, Karolinska Institutet; Örjan Sundin, Department of Psychology and Social Work, Mid Sweden University; Runa Sundelin, Department of Clinical Science and Education, Karolinska Institutet and Department of Cardiology Södersjukhuset; Philip Leissner, Department of Women’s and Children’s Health, Uppsala University; Lena Kövamees, patient representative, Swedish Heart and Lung Association; Per Tornvall, Department of Clinical Science and Education, Karolinska Institutet and Department of Cardiology Södersjukhuset
Name and contact information for the trial sponsor {5b}	Per Tornvall, Karolinska Institutet, per.tornvall@ki.se
Role of sponsor {5c}	The funding agency did not take part in the study design, or data collection or any other part of the trial. The sponsor declares that there is no commercial interest in the trial.

## Introduction

### Background and rationale {6a}

The target diagnoses for the present study are myocardial infarction with non-obstructive coronary arteries (MINOCA) and Takotsubo syndrome (TS). Until recently, TS was included as a sub-category diagnosis of MINOCA, but following the recent 4th revision of the diagnostic definition of myocardial infarction (MI), it was restricted to ischemic causes, thus excluding TS from MINOCA [1]. Approximately 6% (5–15%) of all patients with suspected MI have no obvious cause in the coronary arteries, i.e. < 50% stenosis [2–5]. While MINOCA is considered an MI and is due to various causes, TS presents with similar symptoms as MI but is caused by a transient hypokinesia/akinesia of particularly the apical and midventricular parts of the left ventricle associated with increased sympathetic activity.

Patients diagnosed with MINOCA or TS are more likely to be younger and female and less likely diabetic, hypertensive or dyslipidaemic in comparison to patients diagnosed with obstructive MI. In approximately two-thirds of the cases, mental or physical stress is contributing triggers to the events [6]. Patients with MINOCA or TS report increased anxiety, perceived stress and decreased health-related quality of life (QoL) compared to healthy people [7–9]. Increased mental stress and anxiety in patients with TS has been confirmed in qualitative interview studies [10, 11]. Evidence-based treatment for these two entities is limited. At present, there are no randomised studies of any psychological treatment of MINOCA, or TS. There is thus an urgent need for non-pharmacological alternatives aiming at relieving stress and anxiety in MINOCA and TS. Cognitive behavioural therapy (CBT) aiming at reducing anxiety has been shown to be effective [12]. The current protocol describes a randomised controlled trial evaluating an Internet-based CBT (iCBT) programme for reduction of stress and anxiety in patients with increased mental stress and/or anxiety and a final diagnosis of either MINOCA or TS. The development of the intervention, which was conducted in close collaboration with patient research partners, has been described elsewhere [13]. A study addressing the feasibility of recruitment, data collection and intervention delivery, along with patient acceptability of the intervention, has been conducted but is presently unpublished [14].

### Objectives {7}

The overall aim is to study the effects of an iCBT intervention aiming at reducing mental stress and anxiety in patients with MINOCA and TS compared to controls receiving usual care.

### Trial design {8}

The study is a randomised controlled trial with parallel group design and a superiority framework. Allocation ratio is 1:1 to the treatment group and the control group. The primary timely endpoint is 10 weeks after randomisation. After that time, the control group also receives the treatment, thus becoming what we refer to as the late intervention group (LIG). The treatment group is likewise referred to as the early intervention group (EIG). Comparisons at 20 and 50 weeks after randomisation will be made for the total group in comparison to baseline (at randomisation) and between the EIG and LIG.

## Methods: participants, interventions and outcomes

### Study setting {9}

The study setting is four Academic hospitals in Stockholm, Sweden.

### Eligibility criteria {10}

Inclusion criteria for participation are:

- A final diagnosis of MINOCA or TS with coronary angiography without stenosis ≥ 50% within 1 month of the acute event
- Age 35–80 years
- A Perceived Stress Scale (14-item version; PSS-14) score ≥ 25 and/or a Hospital Anxiety and Depression Scale, anxiety subscale (HADS-A) score ≥ 8 at screening
- Reading and writing proficiency in Swedish
- Computer/Internet access and literacy

Exclusion criteria for participation are:

- Strong clinical suspicion of myocarditis
- Spontaneous coronary artery dissection
- Acute pulmonary embolism
- Acute MI type 2
- Cardiomyopathy other than TS
- A previous myocardial infarction due to coronary artery disease (CAD)
- Expected poor compliance to behavioural therapy
- Not likely to survive > 1 year due to for example cancer
- Suicidal ideation

Eligibility criterion for the personnel supporting the participants, the therapist, in the iCBT is being a licenced psychologist in Sweden.

### Who will take informed consent? {26a}

Participants will be informed both orally and in writing about the study by nurses or doctors at the hospital, during index hospitalisation. Informed consent is obtained at the same time and will be registered in the patients’ medical record.

### Additional consent provisions for collection and use of participant data and biological specimens {26b}

This trial does involve collecting biological specimens — hair and saliva for analyses of cortisol — but these samples will be destroyed immediately and not used in further studies.

### Interventions

#### Explanation for the choice of comparators {6b}

iCBT has been shown to be effective in reducing stress and anxiety in other somatic patient groups, including patients with CAD [15]. No study has so far evaluated any psychological treatment for this patient group. Usual care is therefore the natural comparator as no other alternative is established.

### Intervention description {11a}

The intervention is a 9-step therapist-guided Internet-delivered self-help programme, based on CBT. Its development has been described previously [13]. It will be delivered via a secure on-line platform, the portal, using double authentication for log in. All nine intervention steps include short informative texts and examples, as well as exercises which the participant reports in the portal. The self-help material also includes both audio and video files. Participants will be encouraged to complete one step in the self-help programme each week.

The first step includes written medical information about MINOCA and TS and recorded video interviews with a patient and healthcare personnel. Information on the diagnosis and recommended treatments are covered and common reactions after such a cardiac event are presented. In the first step of the intervention, the participants are asked to describe their cardiac event and how it has influenced their lives.

The following four steps focus on stress. Step 2 is mainly psycho-educative and introduces self-monitoring of stressors and stress reactions. In step 3, specific and personal stress situations are identified, including a discussion on short- and long-term consequences of stress behaviours. Step 4 focuses on recovery and relaxation. In step 5, central personal values are identified and behaviours in line with these values are encouraged.

In step 6, the concept of heart-related fear, worry or anxiety is introduced. Participants recognising this kind of worry are encouraged to work through steps 7–8, including relevant psychoeducation, self-monitoring of avoidance behaviours and heart-related exposure training. Participants can skip these two steps and go directly to step 9.

In the last and 9th step of the programme, participants will do a summary of what they have learnt during the intervention, and how these advances can be maintained or further developed.

On completion of the exercises, participants will receive personalised written feedback from their therapist. The participants will also be able to contact their therapist using a text message function within the portal.

### Criteria for discontinuing or modifying allocated interventions {11b}

At baseline, all participants answer a question about suicidal ideation, i.e. question 9 from the Montgomery-Asberg Depression Rating Scale, self-rating version (MADRS-S) [16]. If participants score higher than 3 (which indicates suicidal ideation), a clinical psychologist will be notified automatically and he or she will conduct a structured suicide assessment with the participant via telephone. This may result in discontinuation from the study and referral to standard healthcare if suicidal ideation is confirmed. During the intervention, participants also do weekly ratings of depression and anxiety with the Patient Health Questionnaire 4-item version (PHQ-4) [17]. In case of an increase, judged by the therapist as serious, he or she will contact the participant via telephone according to the same procedure as described above. This is also the case if suicidal ideation or a worsening of depression is signalled in any other communication. If several (>5) participants are recommended to discontinue their participation because of the intervention’s negative influence on them, modifications or termination of the study will be considered.

### Strategies to improve adherence to interventions {11c}

Patients are approached at hospitals by personnel experienced with psychosocial stress in relation to MINOCA and TS. The participants have regular support from a therapist throughout the programme via the Internet platform. The therapist will call the participants after the first step (usually the first week) of the treatment and again after the third step (third week) to discuss the experience of the treatment, expectations, personal goals and potential difficulties. Participants are encouraged to set a day of the week when they are to report their exercise, and weekly prompts are sent as reminders. If a report is late, the therapist sends additional reminders. If participants have not been active for more than 2 weeks, they will also be called by the therapist. Prompts are sent by sms and e-mail when it is time to submit self-rated outcome data. Results from the feasibility study indicate good adherence to the intervention [14].

### Relevant concomitant care permitted or prohibited during the trial {11d}

All usual care is permitted during the trial for both groups and no particular treatment or activity is disapproved.

### Provisions for post-trial care {30}

There is no anticipated harm or compensation for trial participation.

### Outcomes {12}

#### Primary outcome

Proportion of patients with both normal ratings of self-rated stress (PSS-14 < 25) and anxiety (HADS-A < 8) 10 weeks after randomisation in the intervention group and the control group will be compared.

#### Secondary outcomes

- Proportion of patients with normal ratings of self-rated stress (PSS-14 <25) and anxiety (HADS-A<8) at 20 and 50 weeks after randomisation in the EIG and the LIG will be compared.
- Self-rated continuous measures of self-rated stress, anxiety, QoL, depression, cardiac anxiety and post-traumatic stress symptoms 10 weeks after randomisation in the intervention group and the control group will be compared.
- At 20 and 50 weeks after randomisation, continuous measures of self-rated stress, anxiety, QoL, depression, cardiac anxiety and post-traumatic stress symptoms for all participants will be compared to baseline. At the same time points, the same measures in the EIG and the LIG will be compared.
- Total amount of sick leave from randomisation until 20 and 50 weeks respectively in the EIG and the LIG will be compared.
- Total number of healthcare visits from randomisation until 20 and 50 weeks respectively in the EIG and the LIG will be compared.
- The physiological procedure (the stress test) will be conducted 10 weeks after randomisation. The intervention and control groups will be compared regarding HRV (1) at rest, (2) after stress induction and (3) during relaxation (see details below). Blood pressure and saliva cortisol will be compared between the intervention and control groups (1) at baseline, (2) after the stress induction and (3) after relaxation.
- Hair cortisol 10 weeks after randomisation will be compared between the intervention and control groups. A hair sample is taken at the stress test visit.

### Participant timeline {13}

Patients admitted because of suspected MINOCA or TS are screened for eligibility within 30 days of the event allowing for cardiac magnetic resonance imaging when applicable. The screening is a stepwise process where inclusion criteria including proficiency in Swedish and computer literacy are checked during admission. The stress and anxiety inclusion criteria, using the self-rating questionnaires, may be screened for after discharge, at a polyclinic visit, if the clinical assessment is extended. A hair sample is taken at the same time as the questionnaires.

Two weeks after the screening, the included participants will be asked to log in to the secure Internet platform and there fill in the baseline questionnaires. They are then automatically randomised to either iCBT or usual care. The intervention group get immediate access to the intervention.

Ten weeks after randomisation, data are collected during the first follow-up. This includes the Internet-based questionnaires including questions about healthcare consumption and sick leave, the physiological stress test and the second hair sample. After this follow-up, the control group is given access to the intervention.

**Fig. 1:**
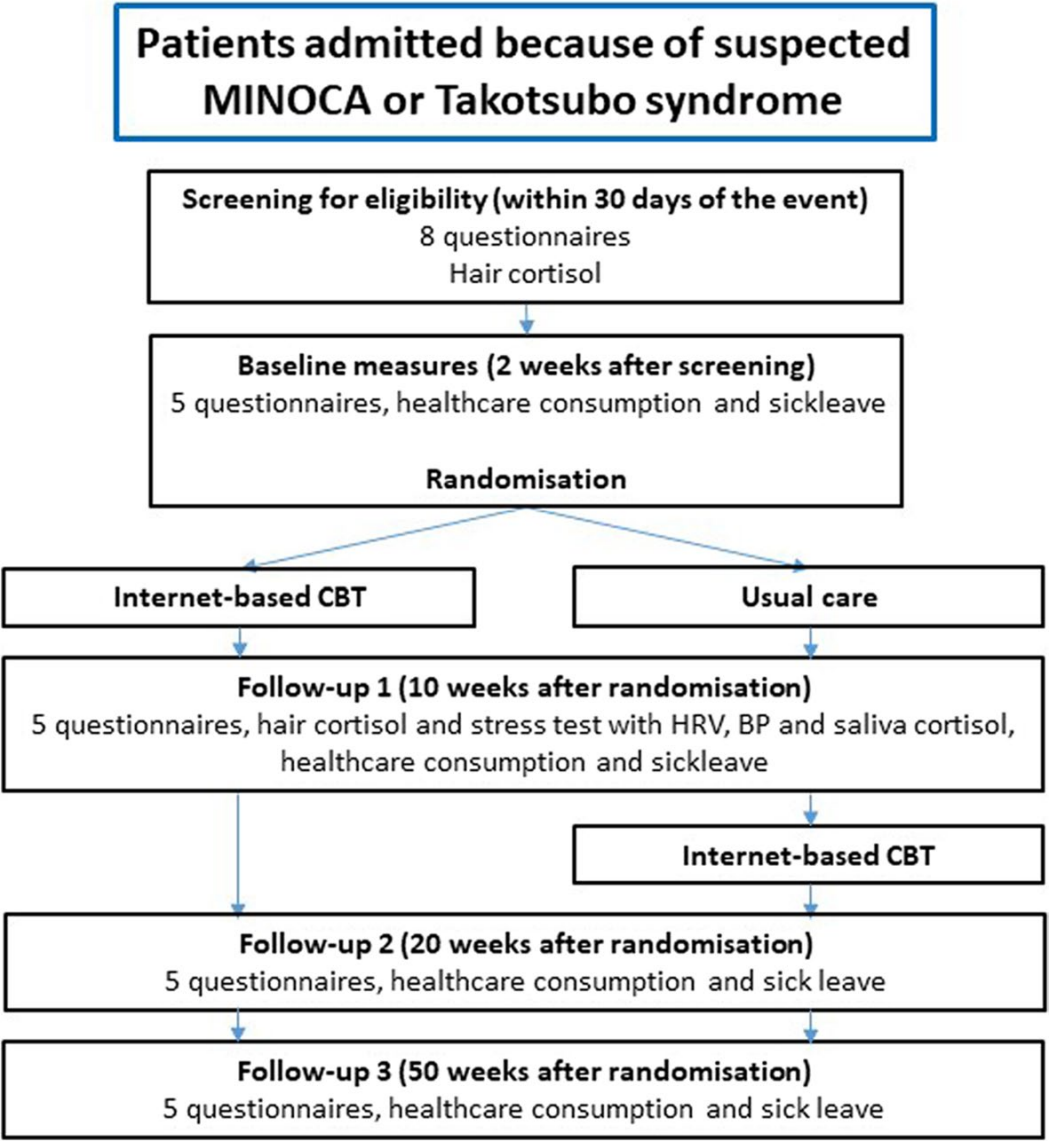
Participant timeline

Twenty and 50 weeks after randomisation, data are collected via Internet-based questionnaire healthcare consumption and sick leave during follow-ups 2 and 3. See Fig. 1 for details.

### Sample size {14}

According to unpublished results from the SMINC-2 study, 45% of the patients receiving usual care normalised both their PSS-14 and HADS-A 6 months after the event. We expect an absolute 30% additional effect of treatment resulting in 75% of the participants in the intervention group normalising both these scores. To detect such a difference with 80% power (*p* < 0.05), 80 participants are needed (40 + 40). By including 90 patients, we will allow for slightly more than 10% attrition.

### Recruitment {15}

Participants will be recruited to the study from four hospitals in Stockholm, Sweden. According to the feasibility study, four hospitals will be needed to reach the participant recruitment target.

## Assignment of interventions: allocation

### Sequence generation {16a}

Randomisation will be made by computer-generated random numbers in blocks of 6 within the Internet portal described above. The randomisation is stratified for study site (hospital).

### Concealment mechanism {16b}

The group allocation code is randomly generated within the Internet portal and totally concealed for the researchers.

### Implementation {16c}

The participants will be asked to log in to the portal and answer baseline questionnaires. After finishing the last item, participants are randomised automatically without involvement of study personnel. The participants allocated to treatment will get immediate access to the intervention on the same portal.

## Assignment of interventions: blinding

### Who will be blinded {17a}

Since this is a study comparing a psychological treatment with treatment as usual, blinding is not possible.

### Procedure for unblinding if needed {17b}

The design is open label so unblinding will not occur.

## Data collection and management

### Plans for assessment and collection of outcomes {18a}

The secure Internet portal described above will be used to collect information about background characteristics and clinical history (including medicine use), as well as for information about healthcare contacts and sick leave in a customized questionnaire. Established and well-validated self-rating instruments will also be used in digital versions on the Internet portal. The physiological stress test is conducted at Södersjukhuset and the ECG data generated during the stress test will be stored on secure computers connected to the equipment. Cortisol and blood pressure data will also be stored on secure computers at Södersjukhuset. See Table 1 for the assessment schedule.

**Table 1:** Assessment schedule

	Screening	Baseline	Follow-up 1	Follow-up 2	Follow-up 3
Demographics and clinical background (self-reported)	X				
Healthcare use and sick leave (self-reported)		X	X	X	X
Perceived Stress Scale, 14-item version	X	X	X	X	X
Hospital Anxiety and Depression Scale	X	X	X	X	X
Montgomery-Asberg Depression Rating Scale, question 9		X			
Rand-36	X	X	X	X	X
Cardiac Anxiety Questionnaire	X	X	X	X	X
Impact of Event Scale, 6-item version	X	X	X	X	X
Intolerance of Uncertainty Scale, 12-item version	X				
Difficulties in Emotion Regulation Scale, 16-item version	X				
Experiences in Close Relationship-Relationships Structure	X				
Physiological stress test (HRV, BP, saliva cortisol)			X		
Hair cortisol	X		X		

Note: *HRV* Heart rate variability, *BP* Blood pressure

### Questionnaires

Self-rated stress will be determined by the PSS-14 which is a self-reported questionnaire measuring levels of stress and coping [18]. PSS-14 contains 14 items that are rated on a 5-point Likert scale, from 0 (never) to 4 (very often) with a total score between 0 and 56. High scores indicate more perceived stress. The threshold for elevated levels of stress is >24. PSS-14 has two subscales where seven of the items measure stress, while the other seven items measure coping ability. The Swedish version of PSS-14 has been shown to be reliable and valid in patients with or without known stress-related conditions [19].

Self-rated anxiety will be determined by the HADS which is an instrument for the assessment of symptoms of anxiety and depression [20]. It has two subscales, one for anxiety (HADS-A) and one for depression (HADS-D), each consisting of seven items rated on a 4-point Likert scale (0–3) with a total score between 0 and 21. A cut-off point of >7 can be used to identify potential cases with clinically relevant levels of anxiety and/or depression. HADS has been validated in a Swedish population and has shown to have good psychometric properties [21]. It has been used frequently in studies focusing on a variety of CAD diagnoses in Sweden and elsewhere.

Self-rated cardiac anxiety will be determined by Cardiac Anxiety Questionnaire (CAQ) which is an instrument used to measure heart-related anxiety developed for patients with chest pain [22]. It consists of three subscales: *fear* of heart-related sensations, *avoidance* of activities that may trigger these sensations and *attention* to these sensations. The scale comprises 18 statements rated on a Likert scale ranging from 0 (never) to 4 (always) resulting in an overall mean score also between 0 and 4. CAQ has demonstrated good reliability and validity and been used in several Swedish patient groups including patients with chest pain with or without CAD and is associated with psychological distress [23].

Self-rated QoL will be determined by Rand-36 which is a free version of the generic QoL questionnaire Short Form-36. It contains 36 items in eight domains of QoL and gives a range between 0 and 100 where a higher value indicates better QoL. The Swedish version of Rand-36 has been validated in cardiac patients [24].

Self-rated post-traumatic symptoms will be determined by Impact of Event Scale, 6-item version (IES-6), which is a short-form version of the Impact of Event Scale, revised version (IES-R) [25]. It is a brief measure capturing symptoms of post-traumatic stress relating to a specified event. The IES-6 includes two IES-R intrusion items, two avoidance items and two hyperarousal items, all rated on a Likert scale ranging from 0 (not at all) to 5 (very much) with a total score between 0 and 30. The IES-6 accounted for 91% of the variance in the full IES-R and possessed screening properties similar to the IES-R when compared to the post-traumatic stress disorder checklist [25]. The Swedish version of IES-R has been shown to have good psychometric properties as a screening tool for post-traumatic stress disorder [26].

Suicidal ideation is determined by a score >3 on question 9 from the Swedish version of the MADRS-S. MADRS-S has shown good psychometric properties [16].

Intolerance of Uncertainty Scale, 12-item version (IUS-12) is a brief self-report tool for evaluating general intolerance of uncertainty [27]. The 12 items are rated on a Likert scale ranging from 1 (not at all characteristic of me) to 5 (entirely characteristic of me). The total score (12–60) indicates general intolerance of uncertainty with higher scores reflecting greater reports of intolerance. The scale can also be divided into two factors: prospective anxiety (fear and anxiety based on future events) comprising 7 items and inhibitory anxiety (uncertainty inhibiting action or experience) comprising 5 items. The English version of the IUS-12 has shown strong internal consistency and high correlations with the original IUS and related measures of anxiety and worry [27], while the Swedish translation is yet to be validated.

Difficulties in Emotion Regulation Scale (DERS-16) is a brief assessment of overall emotion regulation difficulties [28]. It consists of 16 items that assess six dimensions of emotion regulation difficulties: non-acceptance of negative emotions, inability to engage in goal-directed behaviours when distressed, difficulties controlling impulsive behaviours when distressed, limited access to emotion regulation strategies perceived as effective, and lack of emotional clarity. Respondents are asked to rate the extent to which each item applies to them on a 5-point Likert scale from 1 (almost never) to 5 (almost always), giving the DERS-16 a range of 16 to 80, with higher scores reflecting greater levels of emotion dysregulation. The Swedish version of DERS-16 has shown good psychometric properties [28].

Global/general attachment subscale of the Experiences in Close Relationship-Relationships Structure (ECR-RS) questionnaire is an instrument for brief evaluation of an individual’s global adult attachment [29]. In nine items, participants are asked to rate how they generally think and feel in close relationships. Each item is rated on a 7-point Likert scale from 1 (strongly disagree) to 7 (strongly agree). The first six items tap attachment-related avoidance and the three remaining items capture attachment-related anxiety. High scores on either of the two subscales reflect higher levels of attachment-related avoidance and/or anxiety. The Swedish version of ECR-RS has shown good psychometric properties [30].

### Physiological measures

The physiological mental stress test to be described has been used previously in patients with TS and aims at eliciting responses in both the vagal and sympathetic branches of the autonomic nervous system (ANS) and in the hypothalamic-pituitary-adrenal (HPA) axis [31]. The procedure will be conducted approximately 10 weeks after randomisation, i.e. after the intervention. The stress test starts with two stress conditions. The first is an anger recall interview where the patient is asked to recall an upsetting situation and then speak about that situation for 2–3 min. The interviewer asks questions regularly about this upsetting situation to help the participant focus on the situation. The anger recall interview is immediately followed by the second stressor, a mental arithmetic task in which the patient is asked to subtract 7 from a 3-digit number in consecutive steps as fast as possible. The test-leader is repeatedly urging the patient to go faster. The two stress conditions are followed by a recovery phase where the participants are told to relax and imagine a pleasant and secure place or person [32].

Cortisol is a stress hormone and a result of activity in the HPA axis [33]. Measured in saliva, it gives an estimate of the stress response during the last minutes. Saliva cortisol will be used to measure the stress response before and after the two stress conditions. Long-term stress can be indicated by hair cortisol. One centimetre of hair closest to the scalp reflects the most recent month of cortisol exposure. Hair cortisol will be measured at randomisation and after the intervention. Hair and saliva cortisol will be analysed by RIA technique.

Heart rate variability (HRV) reflects normal alterations in the heart rate, i.e. variations in inter-beat intervals, and it reflects ANS activity [34]. HRV is influenced by both vagal and sympathetic nerve activity but in short-term measures the vagal influence dominates. Ten weeks after randomisation, ECG will be recorded for at least 7 min before and after the stress conditions and during the recovery phase. The ECG will be recorded as a single-lead ECG with very high resolution (10,000 Hz; PowerLab, ADInstruments) using a specific electrode positioning that also enables measures of skin sympathetic nerve activity as a surrogate of cardiac sympathetic nerve activity [35]. The following HRV measures will be calculated based on these recordings: in the time-domain as pre-specified outcome measure: root mean square of successive inter-beat interval differences (RMSSD) and standard deviation of normal inter-beat intervals (SDNN); in the frequency domain: high frequency, low frequency and very low frequency HRV, as well as by nonlinear methods (such as Poincaré plots).

Blood pressure will be measured before, during and during the last minute of the two respective stress conditions and during the last minute of the recovery phase.

#### Plans to promote participant retention and complete follow-up {18b}

According to ethical practice, participants can terminate their participation at any point. Participants will be reminded via sms and e-mail if they do not respond within 1 week after being prompted to do so, and again, if still not responding, after 1 week further. Finally, the therapists will call participants that still have not responded and solve any potential hinder.

#### Data management {19}

Self-reported data is entered by the participants directly into the portal database via the Internet. Physiological data will be stored on the computer attached to the equipment before analyses. The final database will be checked manually for erroneous data.

#### Confidentiality {27}

Personal data will be entered through the Internet portal directly by the participants. The personal identification details will be stored in a database separate from all other data. Identifying data are replaced with a study code in the database used for analyses. This code is also used when working with patients in the intervention and study administration. The therapists have access to the personal data necessary to keep a patient record and to contact the participant by phone as stated in the protocol. No one else will access personal data unless absolutely necessary for patient safety reasons, and if so, only by the responsible researcher, or by someone delegated by the responsible researcher. Personal data from recruitment will be stored safely at the respective hospitals.

#### Plans for collection, laboratory evaluation and storage of biological specimens for genetic or molecular analysis in this trial/future use {33}

Hair and saliva will be collected for analyses of cortisol. The samples will be destroyed immediately after analysis and not kept for future analyses.

## Statistical methods

### Statistical methods for primary and secondary outcomes {20a}

Continuous descriptive data will be presented as mean and standard deviation or median with interquartile range and categorical data as frequencies and percentages. Group differences in the primary outcome (normalised ratings on both the PSS-14 and the HADS-A) will be analysed with a logistic regression or a chi-squared test. Group differences in continuous data at all follow-up visits will be analysed with linear regression models, mixed models regression or ANCOVA controlling for baseline measures, sex and age. Longitudinal analyses will be analysed with mixed models regression or ANCOVA. Calculations of effect size will be performed by Cohens’s *d*. The outcomes will be analysed as intention-to-treat with missing data handled by multiple imputation where applicable. Clinical significance will be determined according to the method by Jacobson and Truax [36].

### Interim analyses {21b}

There will be no interim data monitoring since data are self-reported and the patients with active treatment will be followed continuously by psychologists involved in the trial. No problems that are detrimental to the participant are anticipated.

### Methods for additional analyses (e.g. subgroup analyses) {20b}

Complete-case analyses will be done complementary as a sensitivity analysis method.

Exploratory separate subgroup analyses for sex (men and women) and diagnoses (TS and MINOCA) will be performed if possible, depending on subgroup sizes.

Cross-sectional analyses using data at screening will be done to better understand aspects of the distress of patients with MINOCA and TS. These analyses have separate research questions and are not related to the evaluation of the intervention.

### Methods in analysis to handle protocol non-adherence and any statistical methods to handle missing data {20c}

Multiple imputation will be used in the main analysis.

### Plans to give access to the full protocol, participant-level data and statistical code {31c}

The trial protocol is published on clinicaltrials.gov with the reference number 20191111. Access to a case-level dataset is limited due to the General Data Protection Regulation (2016/679). Statistical codes will be available on demand.

## Oversight and monitoring

### Composition of the coordinating centre and trial steering committee {5d}

There will be an audit of the recruitment process by independent monitors from Södersjukhuset at the end of the trial.

### Composition of the data monitoring committee, its role and reporting structure {21a}

There is no data monitoring committee as this is a low-risk intervention.

### Adverse event reporting and harms {22}

Participants will rate their depression and anxiety with the PHQ-4 weekly during the intervention [17]. In case of an increase, judged by the psychologist as serious, the therapist will contact the participant via telephone for a more thorough assessment of suicidal ideation and the causes for the worsening of symptoms. Number of cases contacted for this reason will be reported in publications. Levels of anxiety and depression are outcomes and assessed at every follow-up in the study and will be reported. No other measures of adverse events will be used.

### Frequency and plans for auditing trial conduct {23}

There will be no interim monitoring of the compliance to trial procedures. There will be an audit of the recruitment process by independent monitors from Södersjukhuset at the end of the trial.

### Plans for communicating important protocol amendments to relevant parties (e.g. trial participants, ethical committees) {25}

Changes in the protocol will be reported to the clinicaltrials.gov and to the authority for research ethics who has vetted the study.

## Dissemination plans {31a}

The results of the study will be presented at national and international conferences and published in an open access peer-reviewed journal including authorship by all members of the steering committee and selected doctoral students. One representative from the patient organisation the Swedish Heart and Lung Association is a member of the steering committee. We will present our results to this patient organisation and in their publication for their members.

## Discussion

At present, there are no randomised studies evaluating psychological treatment for patients with MINOCA or TS. The current protocol describes a randomised open-label controlled trial evaluating an iCBT programme for reduction of stress and anxiety in patients with increased mental stress and/or anxiety and either MINOCA or TS.

MINOCA is a relatively new diagnosis and it is directly dependent on criteria for MI. This means that previous studies may have included different categories of patients, e.g. patients with TS. The diagnosis of MINOCA should be set after a thorough clinical assessments ultimately including cardiac magnetic resonance imaging. Not all hospitals have this as a routine and sometimes it takes some time after the event until this procedure is available. In the present study, we therefore allow for 30 days to confirm the diagnosis and to rule out the clinical exclusion criteria. The advantage thus being an increased validity of eligibility criteria. The disadvantage is that there will be a relatively big variety in the time between the event and randomisation for different participants.

It is well known that the physiological responses to stress are involved in triggering cardiovascular events [37]. In TS, this is well documented and there is also much support for the same mechanisms in MINOCA [6, 38]. Unfortunately, measuring the physiological stress response as a meaningful outcome of an intervention has been proven difficult due to a large individual variability and the difficulty to control for individual differences in perception and appraisal [39]. Therefore, the most valid outcome measures of stress, so far, are self-reported and therefore subjective [18]. There are however relevant and promising physiological measures and procedures that may complement the subjective ratings informing about physiological mechanisms involved in the stress response and stress recovery. In the present study, a procedure loosely inspired by the Trier Social Stress Test, replacing the presentation with an anger recall condition, is used [31, 39]. HRV as a measure of ANS activity, cortisol as an end-product of the HPA axis and BP as a relevant cardiovascular response are all relevant measures. These measures are considered as secondary outcomes as there is still lacking consensus of how to best measure physiological stress as an outcome of behavioural interventions. Any findings may be hypothesis-generating for future studies.

The present study has been preceded by a thorough development phase including collaboration with patient representatives and a feasibility study [13, 14]. The feasibility of recruitment, data collection and the intervention was investigated against progression criteria, and even though the study was conducted during the COVID-19 outbreak, most criteria were met. Moderate challenges with participant recruitment were highlighted. This will likely be a main challenge in the present study, but by including four hospitals, we will likely succeed in recruiting to target within the set time frame.

If the results are positive, a now lacking behavioural treatment option reducing stress and anxiety could be available in the secondary prevention after a MINOCA or TS event in the future.

### Trial status

Recruitment is ongoing since November 2021. Recruitment is anticipated to end by the 31st of December 2023.

## Abbreviations

ANS: Autonomic nervous system
CAD: Coronary artery disease
CAQ: Cardiac Anxiety Questionnaire
CBT: Cognitive behaviour therapy
DERS-16: Difficulties in Emotion Regulation Scale, 16-item version
ECR-RS: Experiences in Close Relationship-Relationships Structure
EIG: Early intervention group
HADS: Hospital Anxiety and Depression Scale
HADS-A: Hospital Anxiety and Depression Scale, anxiety subscale
HPA: Hypothalamic-pituitary-adrenal
HRV: Heart rate variability
iCBT: Internet-based cognitive behaviour therapy
IES-6: Impact of Event Scale, 6-item version
IES-R: Impact of Event Scale, revised version
IUS-12: Intolerance of Uncertainty Scale, 12-item version
LIG: Late intervention group
MADRS-S: Montgomery-Asberg Depression Rating Scale, self-rating version
MI: Myocardial infarction
MINOCA: Myocardial infarction with non-obstructive coronary arteries
PHQ-4: Patient Health Questionnaire, 4-item version
PSS-14: Perceived Stress Scale, 14-item version
QoL: Quality of life
TS: Takotsubo syndrome
